# Influence of the Kinematic System on the Geometrical and Dimensional Accuracy of Holes in Drilling

**DOI:** 10.3390/ma14164568

**Published:** 2021-08-14

**Authors:** Mateusz Bronis, Edward Miko, Lukasz Nowakowski

**Affiliations:** Department of Manufacturing Engineering and Metrology, Kielce University of Technology, al. Tysiaclecia Panstwa Polskiego 7, 25-314 Kielce, Poland; emiko@tu.kielce.pl (E.M.); lukasn@tu.kielce.pl (L.N.)

**Keywords:** drilling, turning center, kinematic system, ANOVA, hole quality

## Abstract

This article attempts to show how the kinematic system affects the geometrical and dimensional accuracy of through-holes in drilling. The hole cutting tests were performed using a universal turning center. The tool was a TiAlN-coated Ø 6 mm drill bit, while the workpiece was a C45 steel cylinder with a diameter of 30 mm and a length of 30 mm. Three kinematic systems were studied. The first consisted of a fixed workpiece and a rotating and linearly moving tool. In the second, the workpiece rotated, while the tool moved linearly. The third system comprised a rotating workpiece and a rotating and linearly moving tool, but they rotated in opposite directions. The geometrical and dimensional accuracy of the hole was assessed by analyzing the cylindricity, straightness, roundness, and diameter errors. The experiment was designed using the Taguchi orthogonal array method to determine the significance of the effects of the input parameters (cutting speed, feed per revolution, and type of kinematic system) on the accuracy errors. A multifactorial statistical analysis (ANOVA) was employed for this purpose. The study revealed that all the input parameters considered had a substantial influence on the hole quality in drilling.

## 1. Introduction

Currently, hole drilling in steels can be performed using a variety of modern methods such as electron beam machining, ultrasound machining, electrical discharge machining, and abrasive water jet machining. Still, it is the conventional drilling methods that are predominant because they are cheap, fast, and simple [1]. Hole cutting is reported to be the most common process performed in the manufacturing sector, representing one third of all machining operations [2,3]. To improve the quality and shorten the time of assembly, engineers make sure the input parameters are properly selected so that high-quality holes are achieved. The assessment of the hole quality generally involves determining the diameter, straightness, roundness, and cylindricity errors [4].

Many studies in this area have aimed at developing more and more accurate models to predict the effects of the drilling process on the hole diameter, roundness and straightness. From the literature, it is evident that few researchers have used their experimental data to create a mathematical model. There are also no studies showing how clamping errors in the case of indexable-insert drills affect the stability of the drilling process and the surface texture of the hole. Such studies have been carried out mainly for milling [5,6]. Aized and Amjad [7], for example, built their models as logarithmic equations to determine how the spindle speed, feed rate, and drilling method (number of steps) affected the hole diameter, roundness, and cylindricity errors. Other researchers [8] looked at the hole diameter and roundness errors as a function of feed rate for three different values of the cutting speed (v_c_ = 7; 24; 28 m/min). Vipin et al. [9] proposed a model for predicting hole diameter errors (HDEs), which takes into account the following input parameters: tool material, spindle speed, feed per revolution, drill bit diameter, and workpiece material. The model proved to be very accurate; the correlation between the predicted values and the observed ones reached 91%. Interesting models for predicting the hole diameter and roundness errors were described in [3]; the models were based on three input parameters: feed rate, spindle speed, and pressure. It was highlighted that the hole diameter was mainly affected by the spindle speed (50.5%). However, the hole roundness error was dependent primarily on the spindle speed (42%). Kurt, Bagci, and Kaynak [10] developed a model for determining the hole diameter accuracy for four input parameters: depth of drilling, drill bit coating type, cutting speed, and feed per revolution. The predictive model was reported to reach an accuracy of 88%. Çiçek, Kivak, and Ekici [11] created a model for predicting roundness errors from three parameters: tool type, cutting speed, and feed per revolution. They found that the combined influence of the cutting speed and the feed per revolution on the hole roundness error was approximately 64%. The investigations presented in [12] concentrated on the effect of the spindle speed on the hole accuracy calculated as a percentage. Singh, Kumar, and Saini [2] discussed the effects of the spindle speed, feed per revolution, and point angle on the hole diameter error. They did not, however, propose any mathematical model based on their experimental data. The research described in [13] was limited to the measurement of the hole diameter at a constant feed rate and two values of the spindle speed (*n* = 3000; 4500 rev/min). No attempt was made to build a predictive model for determining the hole diameter. In another study [14], two input parameters, i.e., the spindle speed (*n* = 600; 1800; 3000 rev/min) and the feed per revolution (f_n_ = 0.04; 0.12; 0.2 mm/rev) were tested; the diameter error was measured for each set of these parameters and for two tool materials. Uçak and Çiçek [15], on the other hand, analyzed the hole diameter errors for two drill bits (uncoated and TiAlN coated) at different types of cooling (no cooling, LN2 cooling, and water cooling). They did not investigate the optimal selection of the process parameters. Other researchers [16,17] measured the hole diameter at the entry and exit for four values of two parameters: spindle speed (*n* = 1000; 3000; 6000; 9000 rev/min) and feed rate (v_f_ = 100; 300; 600; 900 mm/min). Their results indicate that the higher the spindle speed, the higher the hole diameter error at the entry and exit. Still, other studies [1,18] involved determining the effect of the tool coating on the hole diameter error, which was calculated as a function of the number of holes drilled. The effects of the cutting speed, feed per revolution, and kinematic system on the geometrical and dimensional accuracy of holes drilled in 42CrMo4 + QT steel were discussed in the previous article by the authors [19]. The experiments showed that the hole roundness error was mainly dependent on the kinematic system (65%); the influence of the cutting speed and feed per revolution was much smaller (16% and 6%, respectively). Khanna et al. analyzed the behavior of Inconel 718 in cryogenic drilling and dry drilling at constant process parameters (f_n_ = 0.02 mm/rev and v_c_ = 19 m/min), and the output parameters were the hole roundness and cylindricity errors [20]. Sandeep, Ajay, and Jagadesh investigated the effects of graphite, MOS_2_, and Blasocut lubricants on the hole diameter and cylindricity errors [21]. Jia et al. drilled holes using two different drill bits (experimental and original) in Ti alloys as well as CFRP at constant values of the process parameters, and they analyzed the hole diameter and cylindricity errors as a function of the number of holes drilled [22]. Other researchers [23] studied hole drilling in two materials, Ti-6Al-4V and AA7010, for three different sets of cutting parameters (v_c_ = 50; 100; 150 m/min, f_n_ = 0.08; 0.16; 0.24 mm/rev) and measured the hole roundness and diameter errors. Bertolini et al. [24] considered the relationships between the hole diameter and cylindricity errors and five input parameters. They used three different tools (spur drill, coated twist drill, and uncoated twist drill), two types of drilling (dry and cryogenic), four values of the hole depth (z = 2; 4.5; 7; 9.5 mm), two values of the cutting speed (v_c_ = 100; 150 m/min), and three values of the feed per revolution (f_n_ = 0.5; 1; 2 mm/rev). The experiments described in [25] involved measuring the roundness and cylindricity errors for holes drilled with three different drill bits (uncoated, with a diamond-like carbon coating, and with a diamond coating). They used large ranges of the process parameters (*n* = 2000; 3000; 4000; 6000; 8000; 10,000; 12,000; 14,000; 16,000; 18,000 rpm and f_n_ = 0.02; 0.04; 0.08; 0.1; 0.12; 0.15; 0.18; 0.25; 0.3 mm/rev). The research presented in [26] assumed hole drilling at constant process parameters (v_c_ = 80 m/min and f = 100 mm/min) using three different cooling conditions (flood cooling, LN_2_ and LCO_2_). As a result, 146 holes were drilled at the different input parameters; then the roundness and cylindricity errors were measured for each hole. The results were analyzed in groups for every 10 holes.

Considering all of this evidence, it seems that there is no research pertaining to the effects of kinematic systems on the quality of holes drilled in C45 steel. Most studies on the subject focus particularly on the influence of one output parameter, e.g., the diameter or roundness error; they do not take account of cylindricity and straightness errors, which are also important.

The aim of this study was to determine the influence of the input parameters (KIN, v_c_ and f_n_) on the output parameters (CYL, STR, RON and DE) for holes drilled in C45 steel. The percentage contribution of each input parameter was assessed using a multifactorial statistical analysis (ANOVA). The mathematical models built for the output parameters were significant (*p* < 0.05) and the predicted results were in good agreement with the experimental data (R^2^ > 0.8). The kinematic system was reported to have high influence on the hole diameter error (36.61%). The other output parameters, i.e., CYL, RON, and STR, were largely dependent on the feed per revolution.

## 2. Materials and Methods

The specific objective of this study was to establish how the type of kinematic system used for drilling C45 steel contributed to the diameter, roundness, straightness, and cylindricity errors of the holes cut. The testing was carried out using a DMG MORI CTX, Bielefeld, Germany, alpha 500 universal turning center with driven tools. Three kinematic systems were used to perform the drilling.

The tool tested was a 5D drill bit coated with titanium aluminum nitride measuring 6 mm in diameter. The drill bit was clamped in an axial drilling and milling head (VDI30, SAUTER 113180, Metzingen, Germany). Its characteristic features include internal coolant supply and external nozzle. The clamping required using an ER25 DIN 5480 collet chuck, Orion, Ludwigsburg, Germany. One new drill bit was used for each kinematic system. Each tool was used to drill 9 holes. No measurable wear was observed on any of the tools.

The material tested—C45 steel (1.0503)—is a non-alloy quality steel suitable for heat treatment. The chemical composition of the material is provided in Table 1.

The material is easy to work, but difficult to weld. It has high flexural strength, high tensile strength, and high ductility. Table 2 shows the main properties of the steel tested.

**Figure 1:**
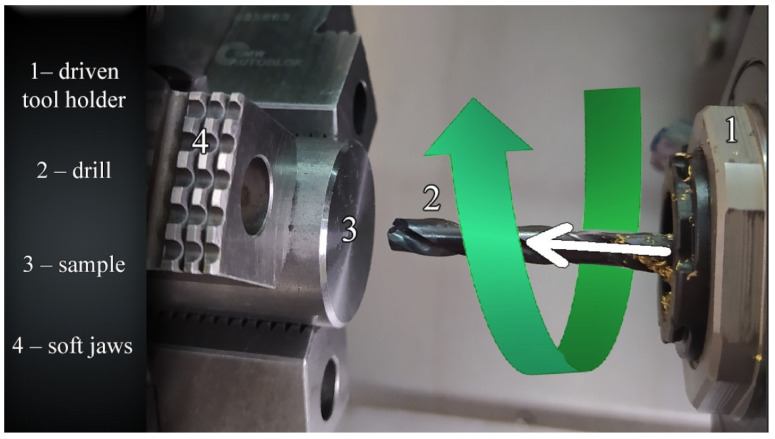
First kinematic system.

**Figure 2:**
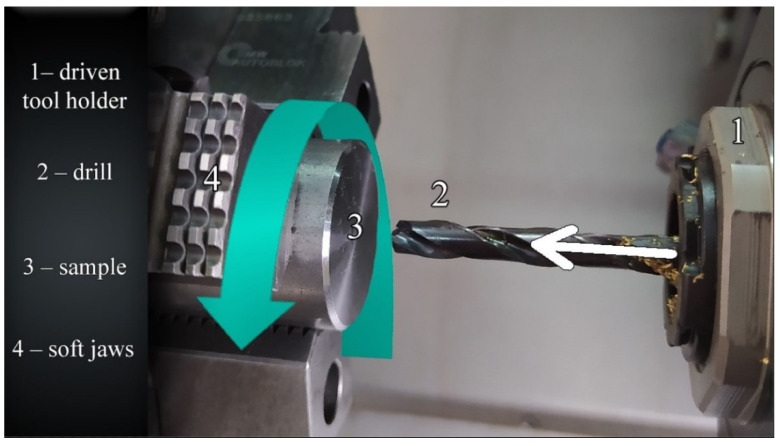
Second kinematic system.

**Figure 3:**
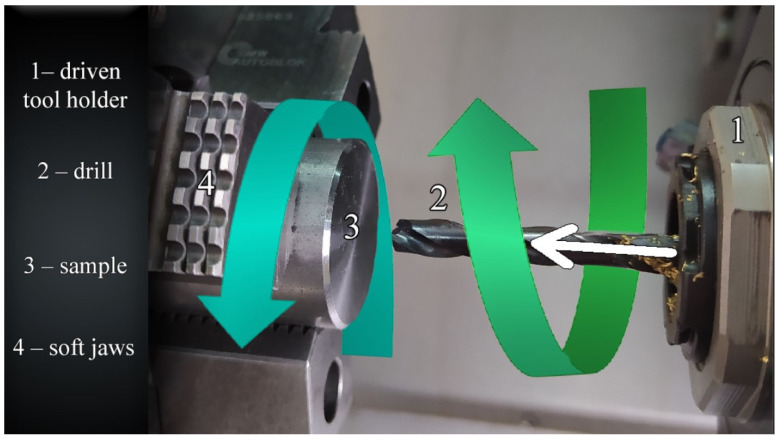
Third kinematic system.

Figure 1, Figure 2 and Figure 3 show the three kinematic systems used for drilling holes. Figure 1 depicts the first kinematic system (KIN I), where the workpiece is fixed and the tool both rotates and moves linearly.

Figure 2 illustrates the second kinematic system (KIN II), where the workpiece rotates and the tool moves linearly parallel to the workpiece axis of rotation.

Figure 3 presents the third kinematic system (KIN III), where the workpiece and tool rotate in opposite directions; the tool also performs a rectilinear motion.

Twenty-seven cylindrical samples 30 mm in diameter and 30 mm in length were used in the drilling tests. They were prepared by planing and clamped in a 3-jaw chuck. The experiments involved drilling 6 mm axial through-holes. Table 3 shows the input parameters and the setting levels.

The drilling was carried out for all the combinations of the parameters presented in Table 3. Thus, 27 holes were cut.

**Figure 4:**
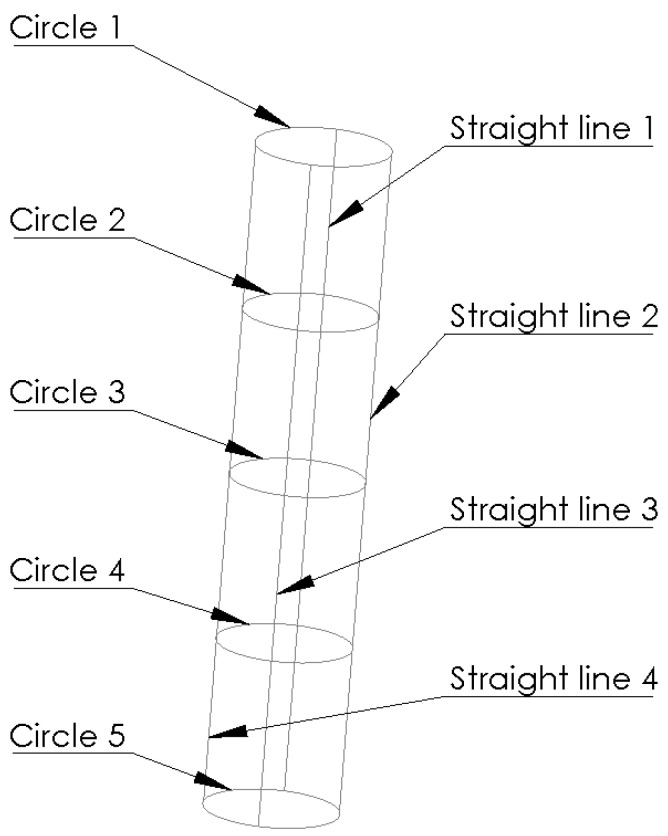
Measurement strategy.

Table 4 shows the input parameters and the corresponding average values of the diameter, roundness, straightness, and cylindricity errors. The metrological results were obtained by means of a ZEISS PRISMO Navigator, Oberkochen, Germany, coordinate measuring machine. The measurements were taken using a ruby probe stylus ball tip with a radius of 1.5 mm at a speed of 5 mm/s. A total of 1500 measuring points were collected. The measurement strategy, illustrated in Figure 4, was as follows. The cylindricity error was established from roundness profiles using a Gaussian filter at 15 UPR and λc = 2.5 mm. The straightness error was measured along the four generatrixes of the hole, spaced every 90°. The diameter and roundness errors were measured at 5 different planes spaced every 7.5 mm, as shown in Figure 4 and indicated as circles, with ‘Circle 1’ and ‘Circle 5’ being the circles measured at the hole exit and entry, respectively.

The experiment required deriving Taguchi L27 orthogonal arrays for three input parameters each at three levels (3 × 3 × 3). Table 4 shows all the sets of the input parameters with their responses.

Statistical calculations were made on the basis of the Taguchi L27 orthogonal arrays. Statistica software (13.3.721.1) was used for this purpose.

## 3. Results and Discussions

The statistical calculations helped assess the effects of the input parameters on the selected output parameters. In each statistical analysis, the confidence level was 95% and the significance level was 5%. Table 5 and Table 6 show the ANOVA results for the particular output parameters. The values of MS and SS provided in Table 5 and Table 6 were used to calculate the value of F, which was then checked in the arrays to determine the significance of the statistical analysis. The analysis indicates that the mathematical models developed for the purpose of this research are significant. The values of *p* were below 0.05, which confirms their significance. As can be seen from Table 6 for the diameter error, the total percentage contribution of the input parameter KIN obtained in the statistical analysis (ANOVA) was 36.61%. The other output parameters were mainly dependent on feed per revolution. For the hole cylindricity error, it was 37.1%. For the straightness error, it reached 30.24%. In the case of the roundness error, the effect of feed per revolution was the highest (81.37%). The mathematical models based on the empirical observations confirm that the correlation between the input and output variables is high (CYL R^2^ = 0.8827, STR R^2^ = 0.9384, RON R^2^ = 0.8584, and DE R^2^ = 0.8369).

The mathematical models built to predict the diameter, roundness, straightness, and cylindricity errors for the material tested were based on the factorial and polynomial response surface regression model. The predictive models are provided in Equations (1)–(4).

(1) $$\begin{array}{c} RON=81.9167-0.7593v_{c}+0.0042{v_{c}}^{2}-8.6236f_{n}+0.3208{f_{n}}^{2}+2.45KIN+0.0167KIN^{2}+0.0147v_{c}\cdot f_{n}\\ \;-0.0222v_{c}\cdot KIN-0.1167f_{n}\cdot KIN \end{array}$$

(2) $$\begin{array}{c} DE=7.1296+0.2774v_{c}-0.0006{v_{c}}^{2}-3.9556f_{n}+0.1403{f_{n}}^{2}+8.9833KIN-0.1722KIN^{2}+0.0006v_{c}\cdot f_{n}\\ \;-0.0867v_{c}\cdot KIN-0.0917f_{n}\cdot KIN \end{array}$$

(3) $$\begin{array}{c} STR=284.8093-2.7704v_{c}+0.0089{v_{c}}^{2}-27.7486f_{n}+0.7972f_{n}{}^{2}+25.9722KIN+1.3889KIN^{2}\\ \;+0.1186v_{c}\cdot f_{n}-0.1222v_{c}\cdot KIN-1.7458f_{n}\cdot KIN \end{array}$$

(4) $$\begin{array}{c} CYL=488.8204-4.8437v_{c}+0.0160{v_{c}}^{2}-44.7736f_{n}+1.2056{f_{n}}^{2}+3.8389KIN+2.9222KIN^{2}+0.1864v_{c}\\ \;\cdot f_{n}-0.0322v_{c}\cdot KIN-0.9250f_{n}\cdot KIN \end{array}$$

where KIN I = 1; KIN II = 2; KIN III = 3.

**Figure 5:**
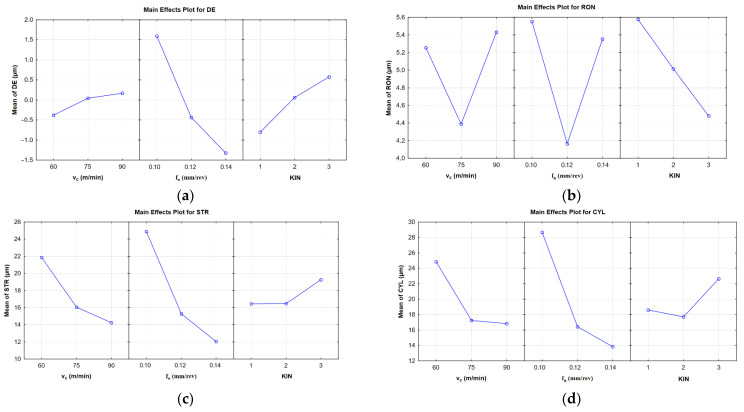
Main effects plots for (**a**) the diameter error; (**b**) the roundness error; (**c**) the straightness error; (**d**) the cylindricity error.

The main effects plots in Figure 5 show the effects of the cutting speed, the feed per revolution and the kinematic system on the diameter, roundness, straightness, and cylindricity errors. From Figure 5a, it is clear that the diameter error decreased with increasing cutting speed; this error also decreased with increasing feed rate. The best results were observed for KIN II: the hole had an ideal diameter of 6 mm. Figure 5b indicates that the lowest values of the roundness error were obtained at the following process parameters: v_c_ = 75 m/min and f_n_ = 0.12 mm/rev. The worst results concerning the roundness error were reported for KIN I (average error: 5.59 µm), while the best for KIN III (average error: 4.49 µm). In the case of KIN II, the average roundness error was 5.03 µm. Figure 5c indicates that an increase in the process parameters (v_c_, f_n_) caused an increase in the average hole straightness error (12 µm for 0.14 mm/rev and 14 µm for 90 m/min). Kinematic systems I and II look similar; for both, the average straightness error was 14.14 µm. However, when KIN III was used, the hole straightness error increased, reaching an average value of 19.15 µm. The same observation was made for the hole straightness error from Figure 5d. The cylindricity error decreased with increasing process parameters (for v_c_ = 90 m/min, the error was 16.89 µm and for f_n_ = 0.14 mm/rev, it was 13.98 µm). Summing up, KIN II had the greatest effect on three out of four output parameters (DE, STR, CYL), which suggests that this kinematic system is the most favorable. However, the hole roundness error was the smallest for KIN III.

Since KIN had the greatest influence on the diameter error (36.61%), as observed in Table 6, the simulations were carried out for each kinematic system to find out how it affected the diameter error.

**Figure 6:**
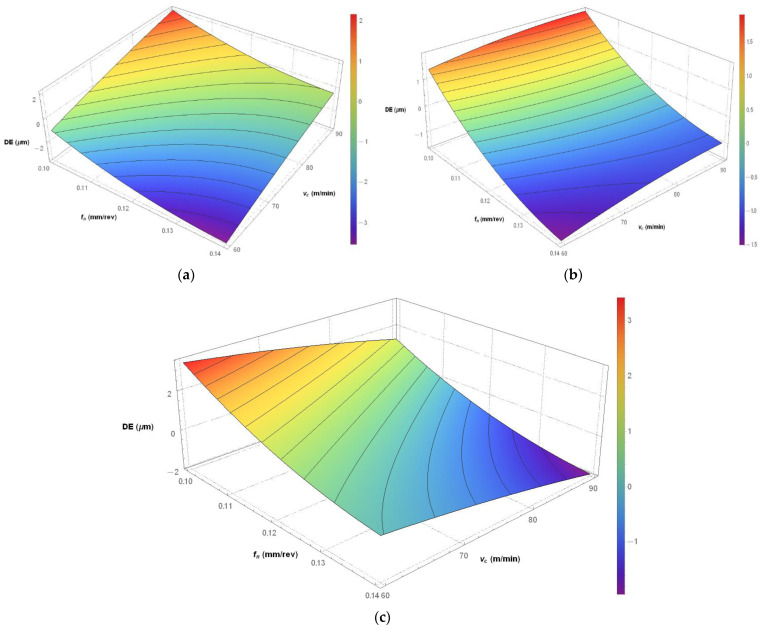
Simulations based on Equation (2) developed to predict the hole diameter error for: (**a**) the first kinematic system (**b**) the second kinematic system; (**c**) the third kinematic system.

The hole diameter error for C45 steel was analyzed with regard to three kinematic systems. From Figure 6a, showing the first kinematic system, it is clear that the highest accuracy in diameter (6 mm) was obtained by linearly increasing both process parameters, i.e., cutting speed and feed per revolution. However, the greatest diameter errors (±2 µm) were reported for two sets of the process parameters, i.e., v_c_ = 90 m/min, f_n_ = 0.1 mm/rev and v_c_ = 60 m/min, f_n_ = 0.14 mm/rev. Figure 6b, depicting the second kinematic system, indicates that at the same process parameters, the accuracy was the lowest and the error reached ± 1.5 µm. For this kinematic system, the most desirable diameter error of 0 µm was achieved at a cutting speed ranging from 60–90 m/min and a feed per revolution of 0.11 mm/rev. It is worth mentioning that the cutting speed did not have a considerable effect on the hole diameter error. However, the other process parameter, i.e., the feed per revolution did contribute to the diameter error. The diameter error increased when the feed per revolution increased above or decreased below 0.11 mm/rev. As can be seen from Figure 6c, there is an inverse relationship between the input parameters and the diameter error for the third kinematic system when compared to the first. The greatest error of ± 1.5 µm was obtained for the following two sets of the process parameters: v_c_ = 60 m/min, f_n_ = 0.1 mm/rev and v_c_ = 90 m/min, f_n_ = 0.14 mm/rev. For this kinematic system, a diameter error of 0 µm was obtained by linearly increasing the cutting speed and decreasing the feed per revolution. The research results can be of practical use to industrial engineers as they will be able to select optimal process parameters to drill a hole with a desired dimensional accuracy.

## 4. Conclusions

The major purpose of this study was to determine how the process parameters as well as the kinematic system used for drilling a hole in C45 steel affected the output parameters such as the diameter, roundness, straightness, and cylindricity errors. The methodology and results described here might be used in practice in the manufacturing sector to optimize the selection of process parameters for hole cutting in this type of steel so that desired values of the hole quality parameters are obtained.

The following are the most important conclusions drawn from the experiment:

- The four mathematical models developed for this study to analyze hole drilling in C45 steel provide a high correlation between the observed values and the predicted ones (for the diameter error, R^2^ = 0.84, for the roundness error, R^2^ = 0.86, for the straightness error, R^2^ = 0.94, and for the cylindricity error, R^2^ = 0.88).
- The type of kinematic system used for drilling in C45 steel is an important factor when the hole diameter is considered.
- For the diameter error, KIN I is a mirror image of KIN III along the diagonal between the v_c_ and f_n_ axes.
- Further research will focus on analyzing the influence of the type of kinematic system on the quality of holes drilled in elements of the jet engine exhaust section.

## Figures and Tables

**Table 1:** Chemical composition of C45 steel, %.

C	Mn	Si	P	S	Cr	Ni	Mo
0.42–0.5	0.5–0.8	max. 0.4	max. 0.045	max. 0.045	max. 0.3	max. 0.3	max. 0.1

**Table 2:** Properties of C45 steel.

Hardness (HB)	Tensile Strength (Rm)	Yield Stress (Re)	Young’s Modulus (E)
≤229	560–850 MPa	275–490 MPa	198–207 GPa

**Table 3:** Input parameters and the setting levels.

Parameters	Settings		
	Level 1	Level 2	Level 3
v_c_ (m/min)	60	75	90
f_n_ (mm/rev)	0.1	0.12	0.14
KIN	1	2	3

**Table 4:** Input parameters and the corresponding output values (calculated average values).

Experiment No	v_c_ (m/min)	f_n_ (mm/rev)	KIN	DE_avg_ (µm)	RON_avg_ (µm)	STR_avg_ (µm)	CYL (µm)
1	60	0.1	1	−2.4	5.8	26.8	35.9
2	60	0.1	2	3.0	6.0	29.8	36.6
3	60	0.1	3	3.6	6.4	44.3	51.8
4	60	0.12	1	−2.4	4.8	20.2	23.8
5	60	0.12	2	−0.8	4.4	17.2	13.5
6	60	0.12	3	0.6	4.6	20.0	18.6
7	60	0.14	1	−3.0	5.8	10.2	12.1
8	60	0.14	2	−1.7	5.1	14.3	11.8
9	60	0.14	3	−0.3	4.3	14.1	19.7
10	75	0.1	1	0.6	5.2	17.8	21.6
11	75	0.1	2	1.3	5.3	20.1	22.1
12	75	0.1	3	2.7	4.4	28.7	27.2
13	75	0.12	1	−1.3	4.5	14.9	16.3
14	75	0.12	2	0.5	3.1	15.6	19.6
15	75	0.12	3	0.0	3.3	14.5	16.8
16	75	0.14	1	−1.7	5.1	14.0	11.4
17	75	0.14	2	−1.4	5.2	10.4	9.5
18	75	0.14	3	−0.4	3.4	8.5	10.7
19	90	0.1	1	3.8	7.0	16.8	17.5
20	90	0.1	2	1.3	5.2	18.4	20.4
21	90	0.1	3	0.4	4.7	21.2	25.0
22	90	0.12	1	−0.4	4.4	10.6	11.4
23	90	0.12	2	−0.7	4.7	11.5	11.4
24	90	0.12	3	0.6	3.7	12.9	16.5
25	90	0.14	1	−0.5	7.6	16.7	17.5
26	90	0.14	2	−1.0	6.1	10.9	14.4
27	90	0.14	3	−2.0	5.5	9.2	17.4

**Table 5:** ANOVA results including the percentage contribution (PC) of the process parameters and the type of kinematic system for the cylindricity and straightness errors.

Parameter	Cylindricity Error						Straightness Error					
Source	SS	DF	MS	F Value	*p* Value	PC	SS	DF	MS	F Value	*p* Value	PC
Model	2042.6497	9	226.9611	14.2082	0.0000	88.27	1475.753	9	163.9725	28.7832	0.0000	93.84
Constant	586.2358	1	586.2358	36.6994	0.0000	25.33	199.0132	1	199.0132	34.9342	0.0000	12.66
v_c_	263.2103	1	263.2103	16.4774	0.0008	11.37	86.1040	1	86.1040	15.1144	0.0012	5.48
v_c_^2^	78.0002	1	78.0002	4.8830	0.0411	3.37	24.1335	1	24.1335	4.2363	0.0552	1.53
f_n_	302.9102	1	302.9102	18.9627	0.0004	13.09	116.3462	1	116.3462	20.4230	0.0003	7.40
f_n_^2^	139.5230	1	139.5230	8.7344	0.0089	6.03	61.0141	1	61.0141	10.7102	0.0045	3.88
KIN	1.8880	1	1.8880	0.1182	0.7352	0.08	86.4200	1	86.4200	15.1699	0.0012	5.50
KIN^2^	51.2363	1	51.2363	3.2075	0.0911	2.21	11.5741	1	11.5741	2.0317	0.1722	0.74
$v_{c}\cdot f_{n}$	375.2008	1	375.2008	23.4882	0.0002	16.21	151.9408	1	151.9408	26.6712	0.0001	9.66
$v_{c}\cdot KIN$	2.8033	1	2.8033	0.1755	0.6805	0.12	40.3333	1	40.3333	7.0800	0.0165	2.56
f_n_·KIN	41.0700	1	41.0700	2.5711	0.1273	1.77	146.3008	1	146.3008	25.6812	0.0001	9.30
Error	271.5577	17	15.9740	—	—	11.73	96.8457	17	5.6968	—	—	6.16
Total	2314.2074	26	—	—	—	100	1572.5985	26	—	—	—	100

**Table 6:** ANOVA results including the percentage contribution (PC) of the process parameters and the type of kinematic system for the roundness and diameter errors.

Parameter	Roundness Error						Diameter error					
Source	SS	DF	MS	F Value	*p* Value	PC	SS	DF	MS	F Value	*p* Value	PC
Model	25.4319	9	2.8258	11.4520	0.0000	85.84	71.3111	9	7.9235	9.6947	0.0000	83.69
Constant	16.4634	1	16.4634	66.7212	0.0000	55.57	0.1247	1	0.1247	0.1526	0.7009	0.15
v_c_	6.4674	1	6.4674	26.2104	0.0001	21.83	0.8633	1	0.8633	1.0563	0.3185	1.01
v_c_^2^	5.4150	1	5.4150	21.9454	0.0002	18.28	0.1157	1	0.1157	0.1416	0.7113	0.14
f_n_	11.2369	1	11.2369	45.5401	0.0000	37.93	2.3642	1	2.3642	2.8927	0.1072	2.77
f_n_^2^	9.8817	1	9.8817	40.0475	0.0000	33.35	1.8891	1	1.8891	2.3114	0.1468	2.22
KIN	0.7690	1	0.7690	3.1165	0.0955	2.60	10.3388	1	10.3388	12.6500	0.0024	12.13
KIN^2^	0.0017	1	0.0017	0.0068	0.9355	0.01	0.1780	1	0.1780	0.2177	0.6467	0.21
$v_{c}\cdot f_{n}$	2.3408	1	2.3408	9.4867	0.0068	7.90	0.0033	1	0.0033	0.0041	0.9498	0.00
$v_{c}\cdot KIN$	1.3333	1	1.3333	5.4036	0.0327	4.50	20.2800	1	20.2800	24.8135	0.0001	23.80
$f_{n}\cdot KIN$	0.6533	1	0.6533	2.6478	0.1221	2.21	0.4033	1	0.4033	0.4935	0.4919	0.47
Error	4.1947	17	0.2467	—	—	14.16	13.8941	17	0.8173	—	—	16.31
Total	29.6267	26	—	—	—	100	85.2052	26	—	—	—	100

## Data Availability

Data sharing is not applicable.

## Nomenclature

CYL	Cylindricity error (µm)
DE	Diameter error (µm)
DF	Degrees of freedom
DOE	Design of experiment
$f_{n}$	feed per revolution (mm/rev)
KIN	Kinematic system
MS	Mean square
*n*	spindle speed (rpm)
*p*	significance
PC	Percentage contribution
RON	Roundness error (µm)
SS	Sum of squares
STR	Straightness error (µm)
UPR	Undulations per revolution
v_c_	cutting speed (m/min)
$v_{f}$	feed rate (mm/min)
λc	wave length (mm)
